# Supplementary material for: Metabolome and Transcriptome Analyses Reveal the Regulatory Mechanisms of Photosynthesis in Developing Ginkgo biloba Leaves
Source: Int J Mol Sci. 2021 Mar 5;22(5):2601. doi: 10.3390/ijms22052601 (PMC7961846; doi:10.3390/ijms22052601)
Supplement: Supplementary file 1 [file ijms-22-02601-s001.pdf]

Supplementary Materials

Table S1. Difference analysis of metabolite abundance.

Metabolites	Log2 _FC		
	T_1 vs T_4	T_2 vs T_4	T_3 vs T_4
2-Deoxy-D-ribose 1-phosphate	2.28	0.11	0.11
2-oxobutanedioic acid	-0.57*	-0.10	0.00
3-Phosphoglycerate	-0.12	-0.05	-0.18*
3-Phospho-D-glyceroyl phosphate	2.12*	1.87*	1.67*
5-Phosphoribosyl diphosphate	5.39*	1.36*	-0.29
Acetyl-CoA	1.26*	0.73*	0.06
Alpha-D-Glucose	-0.07	0.08	0.12
Arbutin	-1.91*	0.34*	0.00
Arbutin-6P	-3.63*	-3.21*	-1.66*
cis-Aconitate	-2.36*	-1.32*	-0.32
Citrate	1.38*	1.07	-0.20
D-Erythrose 4-phosphate	0.17	0.15	-1.11*
D-Fructose 1,6-bisphosphate	-4.08*	-1.82*	-1.48*
D-Fructose 6-phosphate	-2.67*	-1.96*	-0.39
D-Glucono-1,5-lactone	-1.69*	-1.25*	-0.81*
D-Glucono-1,5-lactone 6-phosphate	-2.06*	-1.53*	0.04
D-Glucose	-1.83*	-0.73*	-0.08
D-Glycerate	-1.84*	-1.33*	-0.39
D-Ribose 5-phosphate	-7.32*	-0.68*	-0.07
Glycerone phosphate	3.43*	2.72*	2.75*
Gluconolactone	-1.97*	-0.64	-0.54
Isocitrate	-3.16*	-0.84*	-2.00*
Malate	-1.41*	-0. 67*	-0.43
Oxalacetic acid	-0.55	-0.11	0.19
Phosphoenolpyruvate	0.22	-2.92*	0.38
Salicin	0.09	0.17	-0.07
Salicin 6-phosphate	0.65	0.65	0.71*
Sedoheptulose 1,7-bisphosphate	1.37*	-1.62*	-0.74*
Sedoheptulose 7-phosphate	-0.36	-0.34	0.77*
Succinyl-CoA	0.83*	0.23	-1.35*
Thiamin diphosphate	5.05*	4.048	1.34

* indicates significant differences ($P < 0.05$, the T_4 samples were used as control)

Table S2. Identification of photosynthetic genes.

Gene_ID	Symbol	Description	Function
Gb_01991	At1g56140	Probable LRR receptor-like serine/threonine-protein kinase At1g56140	photosynthetic regulatory gene
Gb_02578	AHA8	ATPase 10, plasma membrane-type	membrane protein
Gb_03067	RH57	DEAD-box ATP-dependent RNA helicase 57	photosynthetic regulatory gene
Gb_05853	CAB151	Chlorophyll a-b binding protein 151, chloroplastic	membrane protein
Gb_07034	PGK3	phosphoglycerate kinase 3	photosynthetic regulatory gene
Gb_10163	CAB8	Chlorophyll a-b binding protein 8, chloroplastic	membrane protein
Gb_10692	NADP-ME4	NADP-dependent malic enzyme 4, chloroplastic	electron transport
Gb_10787	VMAC1	V-type proton ATPase 16 kDa proteolipid subunit	membrane protein
Gb_11687	CTR1	Serine/threonine-protein kinase CTR1	photosynthetic regulatory gene
Gb_12093	At5g56450	Probable ADP,ATP carrier protein At5g56450	membrane protein
Gb_12253	PSAF	Photosystem I reaction center subunit III, chloroplastic	photosynthetic regulatory gene
Gb_12321	ANT1	ADP,ATP carrier protein 1, mitochondrial	membrane protein
Gb_13915	ALA1	Phospholipid-transporting ATPase 1	electron transport
Gb_14232	TPH1	chloroplast phenylalanine hydroxylase [Pinus taeda]	photosynthetic regulatory gene
Gb_14687	PPDK2	Pyruvate, phosphate dikinase 2	electron transport
Gb_15276	LPA2	Protein LOW PSII ACCUMULATION 2, chloroplastic	photosynthetic regulatory gene
Gb_16477	RBCS	Ribulose bisphosphate carboxylase small chain clone 512	photosynthetic regulatory gene
Gb_18791	ATPA	ATP synthase subunit alpha, chloroplastic	electron transport
Gb_20683	At5g60760	P-loop NTPase domain-containing protein LPA1 homolog 1	electron transport
Gb_21134	LHCB7	Chlorophyll a-b binding protein 7, chloroplastic	electron transport
Gb_21299	PSAO	Photosystem I subunit O	photosynthetic regulatory gene
Gb_21309	ACA5	Calcium-transporting ATPase 5, plasma membrane-type	electron transport
Gb_22148	HMA5	Probable copper-transporting ATPase HMA5	electron transport
Gb_22296	PSBY	Photosystem II core complex proteins psbY, chloroplastic	photosynthetic regulatory gene
Gb_22924	TLP40	Peptidyl-prolyl cis-trans isomerase, chloroplastic	photosynthetic regulatory gene
Gb_23487	HPT1	Homogentisate phytyltransferase 1, chloroplastic	photosynthetic regulatory gene
Gb_24499	CRK3	Cysteine-rich receptor-like protein kinase 3	photosynthetic regulatory gene
Gb_25335	FD2	Ferredoxin-2, chloroplastic	electron transport
Gb_26546	CTR1	Serine/threonine-protein kinase CTR1	photosynthetic regulatory gene

Gb_27569	GAPC	Glyceraldehyde-3-phosphate dehydrogenase, cytosolic	photosynthetic regulatory gene
Gb_27633	CAP10A	Chlorophyll a-b binding protein CP24 10A, chloroplastic	membrane protein
Gb_34057	ALA1	Phospholipid-transporting ATPase 1	electron transport
Gb_34162	CHLP	Geranylgeranyl diphosphate reductase, chloroplastic	photosynthetic regulatory gene
Gb_34593	PETJ	Cytochrome c6, chloroplastic	electron transport
Gb_35083	PGK1	Phosphoglycerate kinase 1	membrane protein
Gb_35840	CRD1	Magnesium-protoporphyrin IX monomethyl ester [oxidative] cyclase, chloroplastic	photosynthetic regulatory gene
Gb_36099	Os04g0656 100	Plasma membrane ATPase	electron transport
Gb_37015	ATPD	ATP synthase delta chain, chloroplastic	electron transport
Gb_37146	CSP41A	Chloroplast stem-loop binding protein of 41 kDa a, chloroplastic	photosynthetic regulatory gene
Gb_37729	FTSH12	ATP-dependent zinc metalloprotease FTSH 12, chloroplastic	photosynthetic regulatory gene
Gb_39797	PPC	Phosphoenolpyruvate carboxylase	photosynthetic regulatory gene
Gb_39938	At3g03980	NADPH-dependent aldehyde reductase-like protein, chloroplastic	photosynthetic regulatory gene
Gb_40480	AHA11	ATPase 11, plasma membrane-type	membrane protein
Gb_40561	ndhG	NAD(P)H-quinone oxidoreductase subunit 6, chloroplastic	membrane protein
Gb_41161	LHCB3	Chlorophyll a-b binding protein 3, chloroplastic	membrane protein

Table S3. Identification of the genes encoding transcription factors.

Gene_ID	Transcription factor family	Description
Gb_01212	AP2/ERF	Ethylene-responsive transcription factor ERF017
Gb_01374	KAN	Transcription repressor KAN1
Gb_01873	WRKY	WRKY transcription factor 28
Gb_05053	GATA	Male-specific transcription factor M88B7.2
Gb_05469	MYB	Myb family transcription factor PHL7
Gb_05670	NAC	NAC domain-containing protein 78
Gb_07177	DOF	Dof zinc finger protein DOF
Gb_08437	AP2/ERF	Ethylene-responsive transcription factor RAP2-12
Gb_09495	AP2/ERF	Ethylene-responsive transcription factor RAP2-11
Gb_09907	MYB	Transcription factor MYB30
Gb_11735	bZIP	Protein ABSCISIC ACID-INSENSITIVE 5
Gb_12965	AP2/ERF	Ethylene-responsive transcription factor ERF113
Gb_15398	MADS	MADS-box transcription factor GbMADS9
Gb_18885	IDD	Protein indeterminate-domain 5, chloroplastic
Gb_21530	IDD	Protein indeterminate-domain 2, chloroplastic
Gb_22010	SPL	SQUAMOSA promoter-binding protein-like protein
Gb_22761	ATHB	Class III homeodomain-leucine zipper protein C3HDZ2
Gb_25814	MYB	Putative R2R3-Myb transcription factor
Gb_26822	PHR	Protein PHOSPHATE STARVATION RESPONSE 1
Gb_28483	MYB	Syringolide-induced protein 1-3-1B
Gb_29263	AP2/ERF	Transcription factor AP-2-alpha
Gb_29528	bHLH	Transcription factor TCP8
Gb_32055	WRKY	Probable WRKY transcription factor 20
Gb_35789	TFIIIA	Transcription factor IIIA
Gb_35959	ATHB	homeodomain leucine zipper protein HDZ3, partial
Gb_37689	S1FA	DNA-binding protein S1FA
Gb_38828	CRC	Protein tesmin/TSO1-like CXC 7
Gb_39786	ARF	Auxin response factor 18

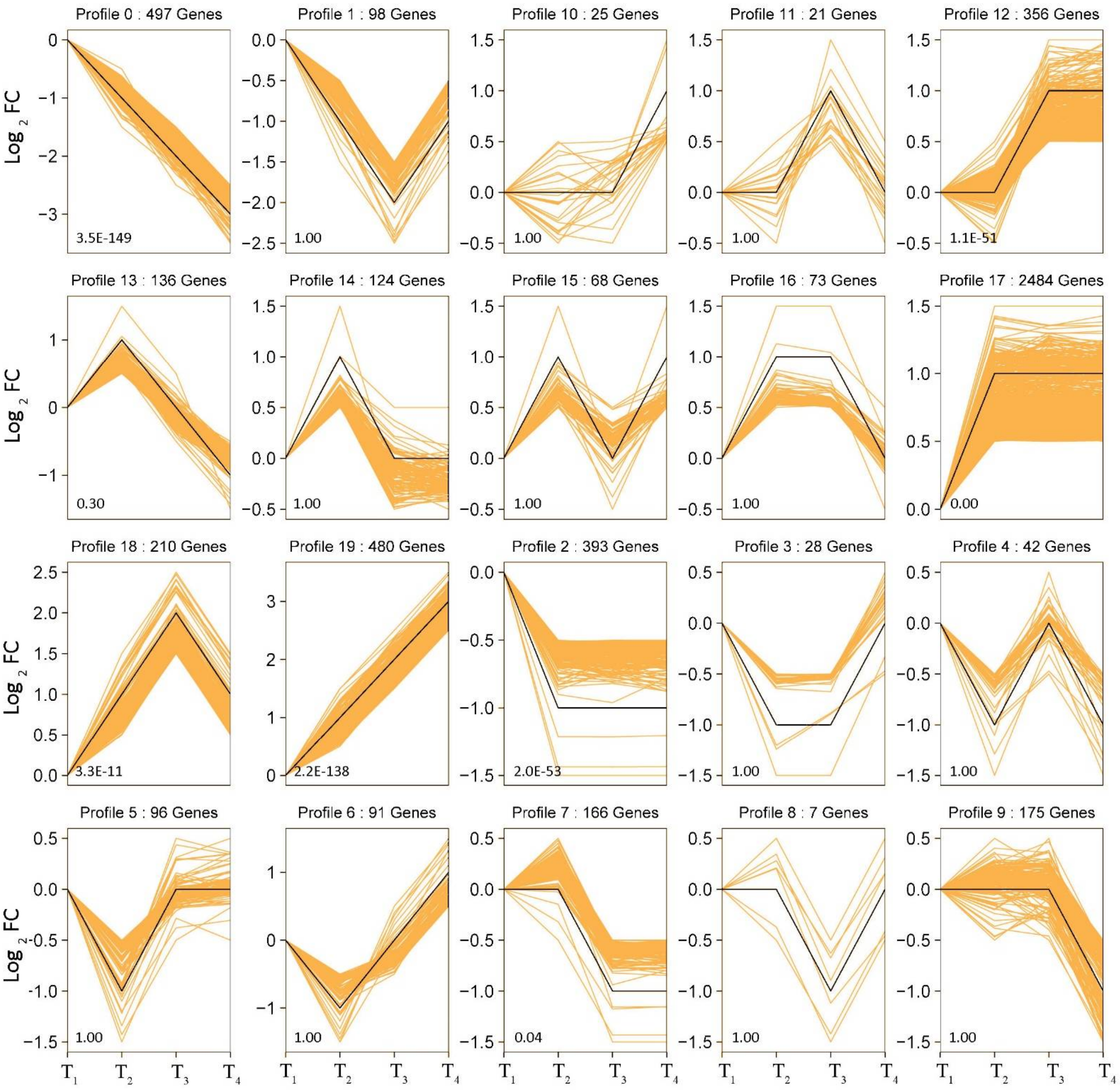

Figure S1. The dynamic expression pattern of differentially expressed genes (DEGs) grouped in 20 clusters based on the similarity of their abundance profiles. Numbers inside Figures represent the *P*-values and black and yellow lines represent the average and each gene's expression trend, respectively.

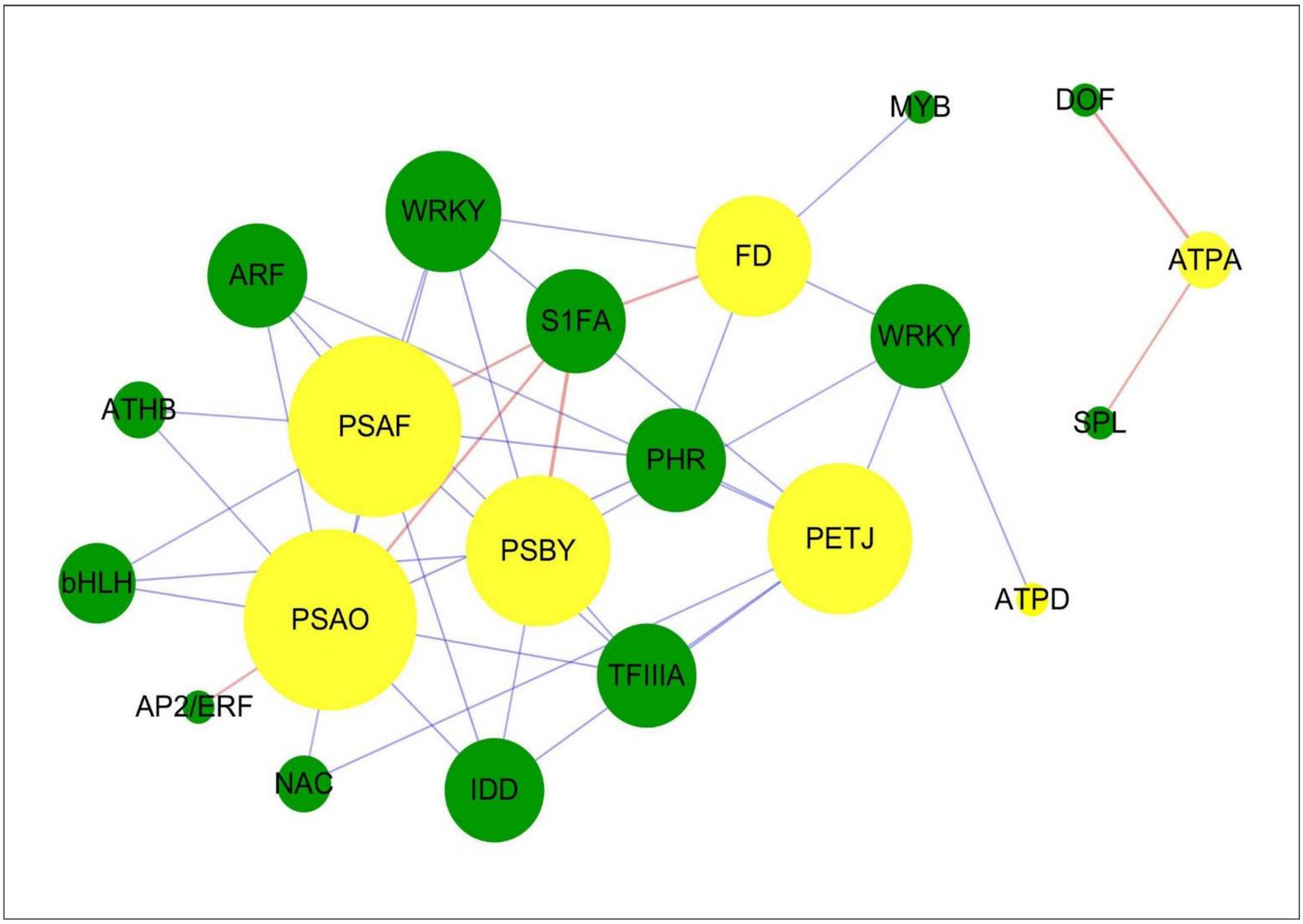

Figure S2. The co-expression sub-network between TFs (green circles) and photosynthetic genes (yellow circles) (circle size is positively correlated with the connectivity of genes in the regulatory network and red and blue lines indicate positive and negative correlations between genes (*P* <0.05, |
